# Relationship between metamorphopsia and inner retinal microstructure following intravitreal ranibizumab injection for branch retinal vein occlusion

**DOI:** 10.1038/s41598-021-84038-7

**Published:** 2021-02-24

**Authors:** Yoshimi Sugiura, Fumiki Okamoto, Tomoya Murakami, Shohei Morikawa, Takahiro Hiraoka, Syed Amal Hussnain, Tetsuro Oshika

**Affiliations:** 1 Department of Ophthalmology, Faculty of Medicine, University of Tsukuba, 1-1-1 Tennoudai, Tsukuba, Ibaraki 305-8575 Japan; 2 Vitreous Retina Macula Consultants of New York, New York, New York USA; 3 Saratoga Ophthalmology, Malta, NY USA

**Keywords:** Retinal diseases, Vision disorders

## Abstract

To evaluate the effects of intravitreal ranibizumab injection (IVR) on metamorphopsia in patients with branch retinal vein occlusion (BRVO), and to assess the relationship between metamorphopsia and inner retinal microstructure and other factors. Thirty-three treatment-naïve eyes of 33 patients with macular edema caused by BRVO with at least 12 months of follow-up were included. The degree of metamorphopsia was quantified using the M-CHARTS. Retinal microstructure was assessed with spectral-domain optical coherence tomography. Disorganization of the retinal inner layers (DRIL) at the first month after resolution of the macular edema (early DRIL) and at 12 months after treatment (after DRIL) was studied. Central retinal thickness (CRT), and status of the external limiting membrane as well as ellipsoid zone were also evaluated. IVR treatment significantly improved best-corrected visual acuity (BCVA) and CRT, but the mean metamorphopsia score did not improve even after 12 months. Post-treatment metamorphopsia scores showed a significant correlation with pre-treatment metamorphopsia scores (*P* < 0.005), the extent of early DRIL (*P* < 0.05) and after DRIL (*P* < 0.05), and the number of injections (*P* < 0.05). Multivariate analysis revealed that the post-treatment mean metamorphopsia score was significantly correlated with the pre-treatment mean metamorphopsia score (*P* < 0.05). IVR treatment significantly improved BCVA and CRT, but not metamorphopsia. Post-treatment metamorphopsia scores were significantly associated with pre-treatment metamorphopsia scores, the extent of DRIL, and the number of injections. Prognostic factor of metamorphopsia was the degree of pre-treatment metamorphopsia.

## Introduction

Macular edema (ME) is a major cause of visual decline following branch retinal vein occlusion (BRVO)^1^. Although visual acuity decreases in the acute phase after BRVO, most patients show significant gains in vision after resolution of ME^2–4^.

Previous reports have shown that ME associated with BRVO affects not only visual acuity, but also vision-related quality of life^5,6^, metamorphopsia^7–11^, and reading speed^12^.

We previously reported that 79.2% patients with treatment-naïve BRVO had metamorphopsia. The metamorphopsia score did not improve by intravitreal ranibizumab injection (IVR) for 6 months, even though visual acuity and central retinal thickness were significantly improved^10^. Osaka et al. also reported that metamorphopsia had persisted after 12 months even when all included eyes achieved reduction in ME and further visual acuity recovery^11^.

Our previous study demonstrated that post-treatment metamorphopsia showed a significant correlation with duration of symptoms, pre-treatment metamorphopsia, and post-treatment disruption of the external limiting membrane (ELM)^10^. Osaka et al. also investigated the correlation with inner, outer, foveal, and total retinal thicknesses, and reported that there was a relationship between post-treatment metamorphopsia and foveal thickness^11^.

Murakami et al. indicated that the presence of inner retinal cystoid change and central retinal thickness were significantly associated with metamorphopsia in BRVO^8^. Other studies have revealed that metamorphopsia is associated with inner retinal thickness in the epiretinal membrane^13,14^ and with fluid cuff in macular hole^15,16^.

Recently, some reports have described that the length of disorganization of the retinal inner layers (DRIL) could be a prognostic factor for visual acuity in patients with diabetic macular edema^17,18^, retinal vein occlusion^19–21^, and uveitic ME^22^. To the best of our knowledge, no previous report has quantified metamorphopsia and inner retinal microstructure including DRIL in patients with BRVO.

This study was designed to evaluate the effects of IVR on metamorphopsia in patients with BRVO and to assess the relationship between metamorphopsia and inner retinal microstructure and other factors (better to list other factors that were studied).

## Results

We included 33 eyes of 33 patients with ME caused by BRVO who were followed monthly for 12 months after initial treatment and were treated with IVR on a pro re nata (PRN) basis. All patients were treatment-naïve with no history of treatment with intravitreal anti-vascular endothelial growth factor (VEGF) injection, intraocular corticosteroids, retinal photocoagulation, or pars plana vitrectomy. We also excluded patients with a previous history of ophthalmic disorders except for mild refractive errors and mild cataracts. We included 15 men and 18 women, averaging 67.2 ± 9.3 years of age (mean ± SD).

**Figure 1:**
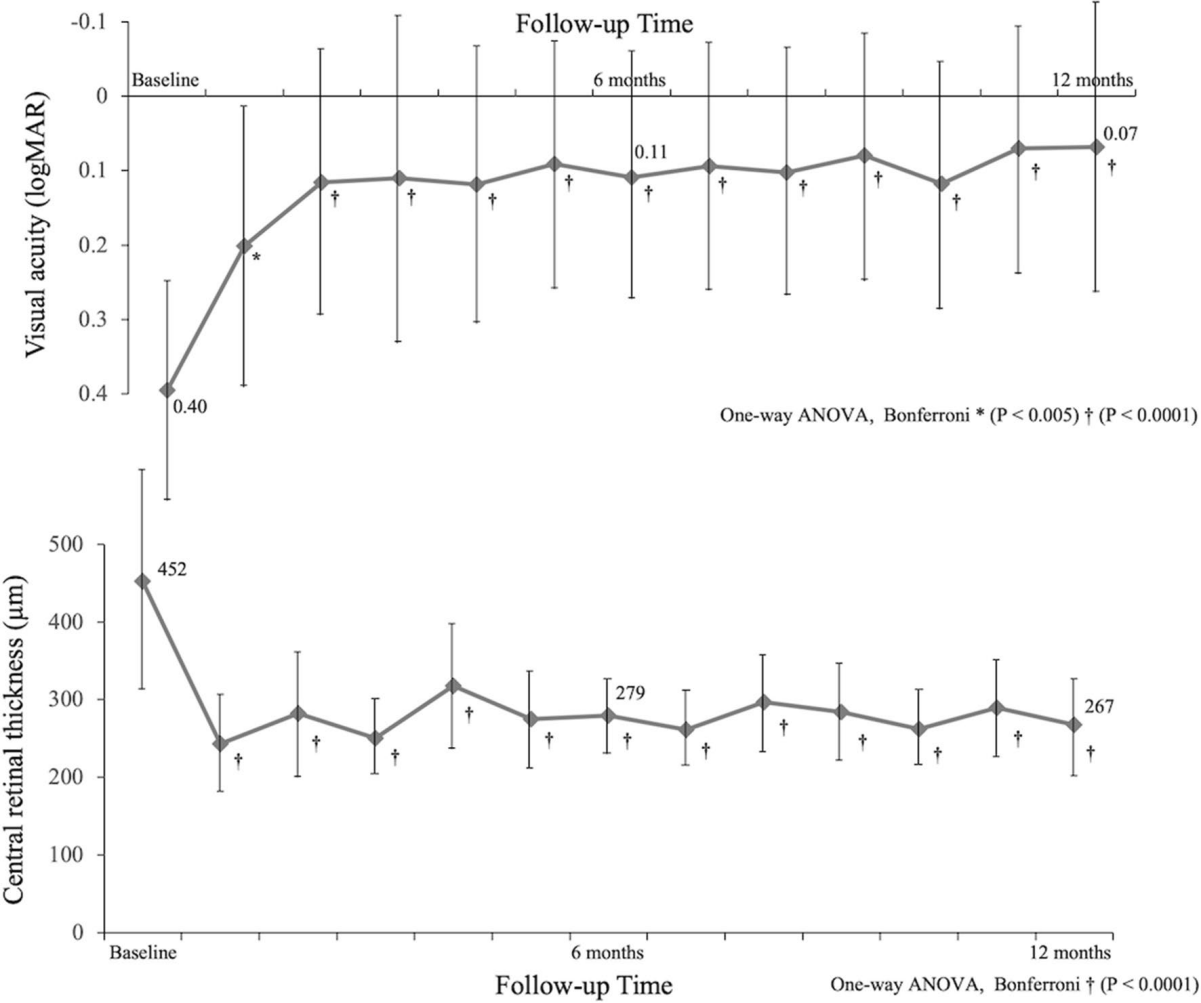
(Top) Time course of changes in visual acuity in patients with branch retinal vein occlusion (BRVO) for 12 months. Intravitreal ranibizumab injection (IVR) treatment significantly improved visual acuity. (Bottom) Time course of changes in central retinal thickness in patients with BRVO for 12 months. IVR treatment significantly improved central retinal thickness from 1 month after treatment.

**Figure 2:**
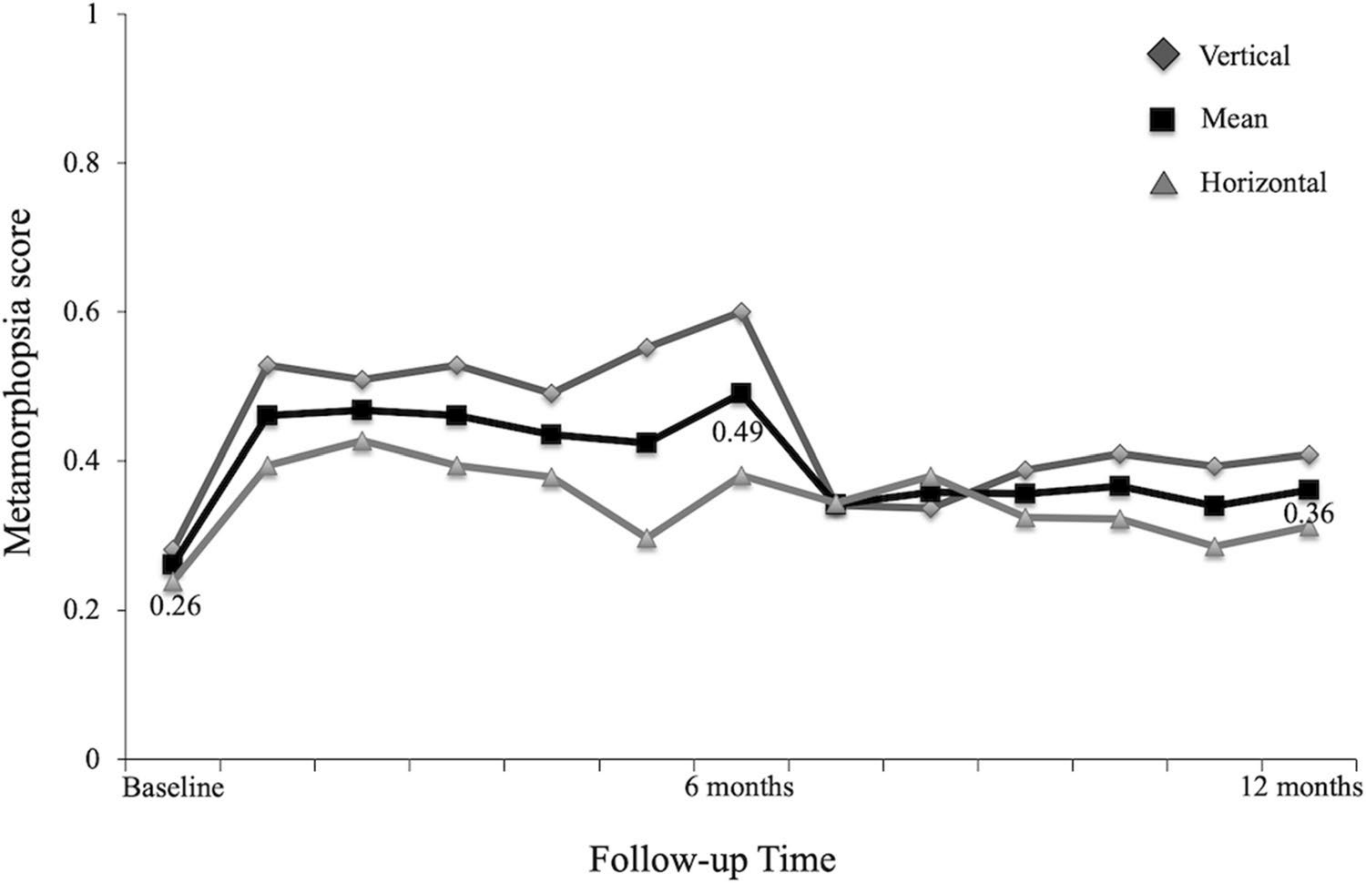
This is time course of changes in metamorphopsia in patients with branch retinal vein occlusion (BRVO) for 12 months. The mean metamorphopsia score did not improve with treatment (*P* = 0.713) during 12 months. Similarly, vertical and horizontal metamorphopsia scores did not improve. Vertical metamorphopsia scores tended to be higher than horizontal metamorphopsia scores, but no significant difference was observed during the follow-up period.

Table 1 shows the clinical characteristics, visual functions, and retinal microstructure before treatment and 12 months after treatment in BRVO patients. There were 29 patients with Major BRVO, and 4 patients with Macular BRVO. Pre-treatment mean metamorphopsia score was 0.26 ± 0.24, with 23 of 33 patients (69.7%) having metamorphopsia (metamorphopsia score ≥ 0.10). Twelve patients had serous retinal detachment (SRD) before treatment, but none patients showed SRD at 12 months after treatment. Figure 1 and 2 show the time course of changes in visual functions and retinal microstructure of patients with BRVO. IVR treatment significantly improved best-corrected visual acuity (BCVA) and central retinal thickness (CRT) (both *P* < 0.0001) (Fig. 1), but the mean metamorphopsia score did not improve with treatment (*P* = 0.713) during 12 months (pre-treatment; 0.26 ± 0.24, 12 months post-treatment; 0.36 ± 0.41) (Fig. 2). Similar results were observed in vertical and horizontal metamorphopsia scores. Pre-treatment and post-treatment vertical metamorphopsia scores were almost higher than horizontal metamorphopsia scores, but no significant difference was observed during the follow-up period. The length of early DRIL was 2601 µm, and the length after treatment was 1616 µm after treatment. There was not significant improvement following IVR treatment (*P* = 0.147).

**Table 1:** Clinical characteristics and time course of changes in visual functions and retinal microstructure following intravitreal ranibizumab injection in patients with branch retinal vein occlusion.

	Pre-treatment	12 months post-treatment	*P* value
Age (years)	67.2 ± 9.3	–	–
Gender (Male/female)	15/18	–	–
Duration of symptoms (months)	2.4 ± 2.3	–	–
Type (major BRVO/macular BRVO)	29/4	–	–
LogMAR BCVA (Snellen visual acuity)	0.40 ± 0.32 (20/50)	0.07 ± 0.24 (20/21)	< 0.0001*
**Metamorphopsia**			
Vertical metamorphopsia score	0.28 ± 0.31	0.41 ± 0.45	0.299
Horizontal metamorphopsia score	0.24 ± 0.22	0.31 ± 0.43	0.957
Mean metamorphopsia score	0.26 ± 0.24	0.36 ± 0.41	0.713
Central retinal thickness (µm)	452 ± 182	267 ± 65	< 0.0001*
Presence of the serous retinal detachment (+ /−)	12/21	0/33	–
Length of DRIL (µm)	2601 ± 1780 (early)	1616 ± 866	0.147
Number of injections	–	4.2 ± 1.4	–

*BRVO* branch retinal vein occlusion, *LogMAR* logarithm of minimal angle of resolution, *BCVA* best-corrected visual acuity, *DRIL* disorganization of the retinal inner layers.

Values are presented as the mean ± standard deviation.

*Significant correlations found between the parameters (One-way analysis of variance and Bonferroni test).

Table 2 shows correlations between 12 months post-treatment visual functions and various parameters. The post-treatment mean metamorphopsia score was significantly and positively correlated with the pre-treatment mean metamorphopsia score, the number of injections (positive), the length of early DRIL (positive), and post-treatment DRIL (positive). The post-treatment BCVA was significantly correlated with the type of BRVO, the pre-treatment BCVA (positive), and the post-treatment disruption of the ELM and the ellipsoid zone (EZ).

**Table 2:** Correlation between 12 months post-treatment visual functions and various parameters in patients with branch retinal vein occlusion (*P* value).

Parameters	Post-treatment mean metamorphopsia score	Post-treatment logMAR BCVA
**Pre-treatment**		
Duration of symptoms	0.263 (rs = 0.195)	0.253 (rs = 0.188)
BRVO type (major/macular )	0.182	0.020^†^
LogMAR BCVA	0.099 (rs = 0.279)	0.001* (rs = 0.524)
Mean metamorphopsia score	0.003* (rs = 0.514)	0.433 (rs = − 0.129)
Central retinal thickness	0.303 (rs = 0.185)	0.479 (rs = 0.120)
Presence of the serous retinal detachment	0.360	0.546
Length of early DRIL	0.035* (rs = 0.379)	0.102 (rs = 0.277)
Number of injections	0.025* (rs = 0.390)	0.277 (rs = 0.189)
**Post-treatment (12 months)**		
Central retinal thickness	0.040* (rs = 0.352)	0.015* (rs = − 0.404)
Length of post-treatment DRIL	0.047* (rs = 0.427)	0.564 (rs = − 0.095)
Disruption of ELM	0.180	0.018^†^
Disruption of EZ	0.397	0.013^†^

*BRVO* branch retinal vein occlusion, *LogMAR* logarithm of minimal angle of resolution, *BCVA* best-corrected visual acuity, *DRIL* disorganization of the retinal inner layers, *ELM* external limiting membrane, EZ: ellipsoid zone.

*Significant correlations found between the parameters (Spearman's rank correlation coefficient test).

^†^Significant correlations found between the parameters (Mann–Whitney U test). rs = Spearman rank correlation coefficient.

Multivariate analysis was performed to reveal the most relevant factor of the post-treatment mean metamorphopsia score. The dependent variables for multivariate analysis included the pre-treatment mean metamorphopsia score, the length of early DRIL, the number of injections, post-treatment CRT, and the length of post-treatment DRIL. We found that the post-treatment mean metamorphopsia score was most significantly correlated with the pre-treatment mean metamorphopsia score (*P* < 0.05).

## Discussion

Metamorphopsia is a common occurrence after BRVO with reported rates ranging from 69 to 93% in previous studies^7–11^. In our study, we observed metamorphopsia in 69.7% of the eyes. We also observed mean pre-treatment and post-treatment metamorphopsia scores of 0.26 and 0.36, respectively. These metamorphopsia scores are lower than those of previous reports. In the pre-treatment period, most patients might have lacked awareness of metamorphopsia, as suggested by the relatively low pre-treatment metamorphopsia average score. However, it is known that an M-CHARTS score of 0.3–0.5 would be the threshold for metamorphopsia as a subjective symptom^23^. Therefore, most patients with BRVO in the post-treatment period might be aware of metamorphopsia and its negative effects on their quality of vision in daily life.

In this study, although there were significant improvements in BCVA and CRT after treatment with IVR, metamorphopsia scores did not change in 12 months. We previously reported similar results in a 6 months follow-up study^10^. Osaka et al. reported that metamorphopsia significantly decreased at 1 month (*P* = 0.044), but no further improvement was achieved with 1 year of additional treatment (*P* = 0.173) in BRVO cases^11^. Thus, once metamorphopsia occurs due to retinal morphological changes in BRVO, it tends to persist even after resolution of ME. There were significant correlations between the mean pre- and post-treatment metamorphopsia scores as well as between the pre- and post-treatment BCVA. These results were similar to those of previous studies^9–11,24^.

The post-treatment metamorphopsia score was also significantly and positively correlated with the number of injections. This indicates that the post-treatment metamorphopsia score was influenced by the number of recurrences. As retinal morphological changes occurred with every recurrence, it likely resulted in deterioration of the retinal structure. It is important to note that the patients in our study were treated with IVR on a PRN basis i.e. treatment was only instituted when ME recurred. It can be argued that using a “treat and extend” approach to avoid any recurrence of ME could improve post-treatment metamorphopsia.

The post-treatment mean metamorphopsia score was also significantly correlated with the length of early DRIL and post-treatment DRIL. Recently, there have been numerous reports about the effects of DRIL in patients with various retinal disorders^17–22^. However, to our knowledge, no study has explored the correlation between metamorphopsia and DRIL in BRVO. Osaka et al. showed that post-treatment metamorphopsia score was correlated with the pre-treatment total foveal thickness^11^. In our previous report, we described that the post-treatment metamorphopsia score was correlated with the post-treatment disruption of the ELM^10^. In this study, we show that 'DRIL' also correlates with the post-treatment metamorphopsia. In acute BRVO, Murakami et al. reported that the severity of metamorphopsia was correlated with the presence of inner retinal cysts in patients with acute BRVO^8^. In other reports, Okamoto et al. described that the degree of metamorphopsia was associated with the thickness of the inner nuclear layer in patients with idiopathic epiretinal membrane. They speculated that the changes in the inner nuclear layer might inhibit the normal function of synaptic junctions and lowers photoreceptor sensitivity thereby causing metamorphopsia^14,25^. We previously documented that the severity of metamorphopsia in patients with macular hole was associated with preoperative area of intraretinal cysts within the fluid cuff^26^. We postulated that intraretinal cysts within the fluid cuff distorted the retinal microstructure, and this morphological change led to metamorphopsia. Similar to these reports, bipolar cells, horizontal cells, and amacrine cells in the inner nuclear layer were damaged by cyst-related BRVO, which could prevent the axonal transport of secondary neurons. We considered that these changes caused the metamorphopsia. Therefore, the disorganization of the inner retina i.e. DRIL might influence the severity of metamorphopsia in patients with BRVO. In this study, the post-treatment metamorphopsia score was not correlated with the post-treatment disruption of the ELM, and there was a discrepancy between this result and the previous result of the 6-month follow-up. In this study we followed up the patients for 12 months, thus increasing the possibility to find cases of improved ELM. Since the main pathology of BRVO was the circulatory disturbance of the retina, the morphologic changes in the inner retina, which are perfused by retinal vessels, might more sensitively reflect the pathological condition.

In this study, we established the correlation between DRIL and metamorphopsia in patients with BRVO for the first time. Previous studies have assessed DRIL with residual ME. However, here we evaluated DRIL after complete resolution of ME. We believe this method gives a more accurate representation of DRIL, as measuring DRIL in the presence of ME likely prevents precise measurements. In the current study, the correlation between post-treatment metamorphopsia and early DRIL suggests that post-treatment visual function can be predicted from early retinal microstructural changes.

We found that metamorphopsia after BRVO is affected by multiple factors, among which pre-treatment metamorphopsia score had the largest impact on the severity of post-treatment metamorphopsia. This suggests that prognosis of metamorphopsia was determined to some extent by initial status in patients with BRVO. However, the number of injections also showed a significant correlation with post-treatment metamorphopsia score. The prognosis of metamorphopsia could likely be improved with a strict treatment regimen after the onset of BRVO.

Our study has several limitations. First, the sample size was small. Second, we evaluated the morphological changes of the retina using SD-OCT, but it is difficult to evaluate the effects of dense retinal hemorrhage, subretinal hemorrhage, hard exudate and ME. It is also necessary to evaluate vision-related quality of life in patients with BRVO, and to assess the correlation between metamorphopsia and quality of life.

In conclusion, metamorphopsia did not change with PRN anti-VEGF treatment over 12 months in patients with BRVO, even when BCVA and CRT were significantly improved. The severity of post-treatment metamorphopsia was significantly associated with the degree of pre-treatment metamorphopsia, the extent of DRIL, and the number of injections. The degree of pre-treatment metamorphopsia was found to be a prognostic factor for metamorphopsia.

## Methods

This was retrospective, observational case series study. All patients were diagnosed with BRVO by fundus examination and spectral domain-OCT (SD-OCT) examination, and confirmed by fluorescein angiography. We classified the patients as 'Major BRVO' or 'Macular BRVO' based on the presence or absence of trunk obstruction in the superior or inferior temporal branches, accompanied by blockage of one or two small branches in the macular area^27,28^.

We also excluded patients with logarithm of minimal angle of resolution BCVA (logMAR BCVA) of > 1.0, because it was impossible to quantify the severity of metamorphopsia correctly^29^. This study was approved by the Institutional Review Board at the Tsukuba University Hospital and was in adherence to the tenets of the Declaration of Helsinki. Signed informed consent was obtained from all study subjects.

We measured BCVA and the severity of metamorphopsia by M-CHARTS (Inami Co, Tokyo, Japan), and the retinal microstructure by SD-OCT (Cirrus high-definition OCT; Carl Zeiss, Dublin, CA). All ophthalmologic examinations were performed at initial presentation and monthly thereafter for 12 months.

BCVA was measured using the Landolt chart and was expressed as the logMAR. The severity of metamorphopsia was assessed using M-CHARTS to quantitatively evaluate the degree of metamorphopsia related to macular diseases^29,30^. It consists of 19 dotted lines with dot intervals ranging from 0.2 to 2.0 degrees of visual angle. If the straight line is substituted with a dotted line, and the dot interval is changed from fine to coarse, the distortion of the line decreases with the increasing dot interval, until the dotted line seems straight. The result is that the greater the degree of metamorphopsia, the higher the M-CHARTS score. First, we showed a vertical straight line (0°) to the patient, and performed the examination. Then the M-CHARTS were rotated 90°, and the same test was performed using horizontal lines. We used vertical and horizontal metamorphopsia scores and their mean score for data analyses. Examinations were repeated three times and were performed at 30 cm so that the refraction of the eye was exactly corrected for this distance.

**Figure 3:**
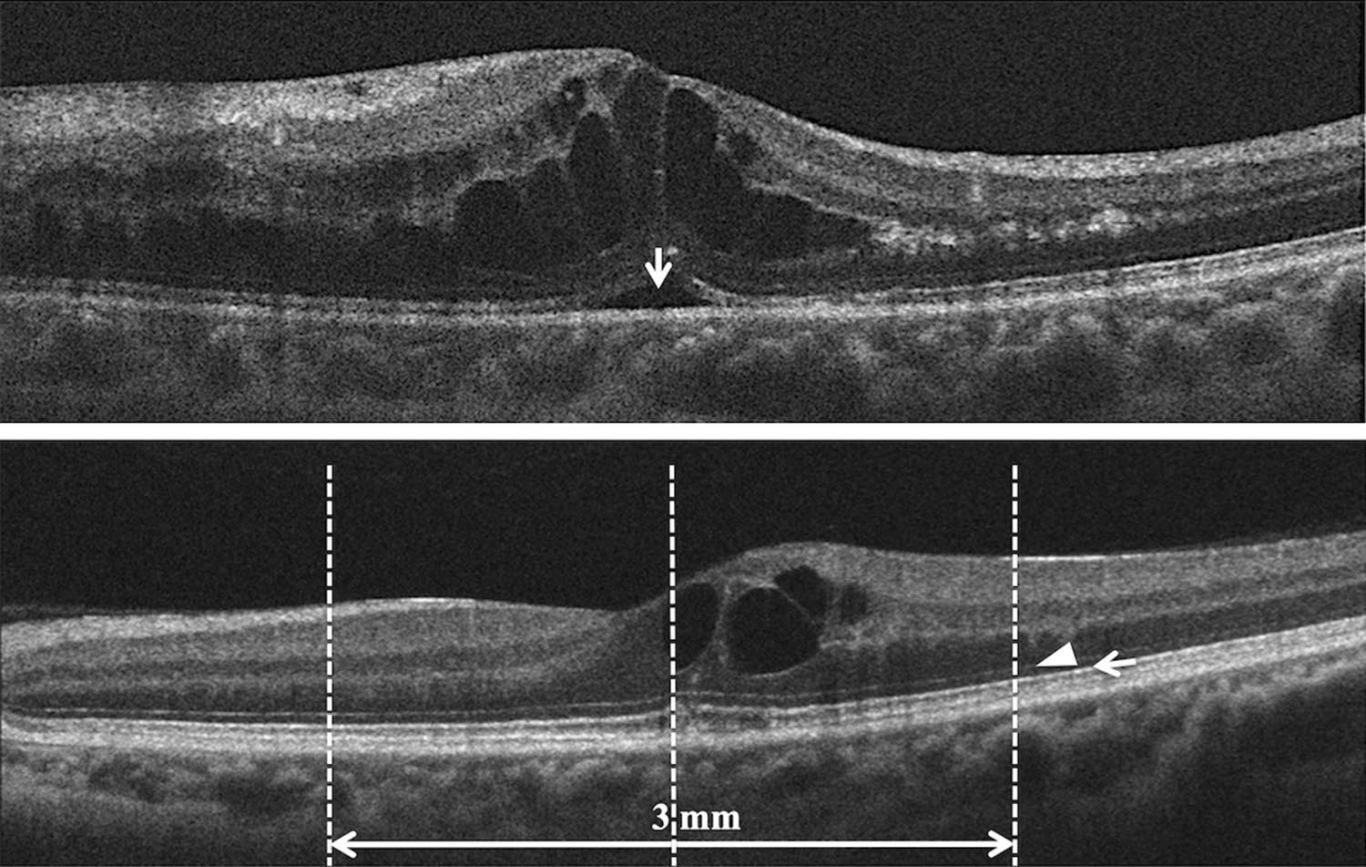
(Top) Based on optical coherence tomography (OCT) images in patients with branch retinal vein occlusion (BRVO), we evaluated the presence of the serous retinal detachment (arrow) before treatment. (Bottom) Based on post-treatment OCT images, we assessed the status (absence or intact) of the external limiting membrane (arrowhead) and the ellipsoid zone (arrow) at the region of 3 mm on the vertical section through the foveal center.

**Figure 4:**
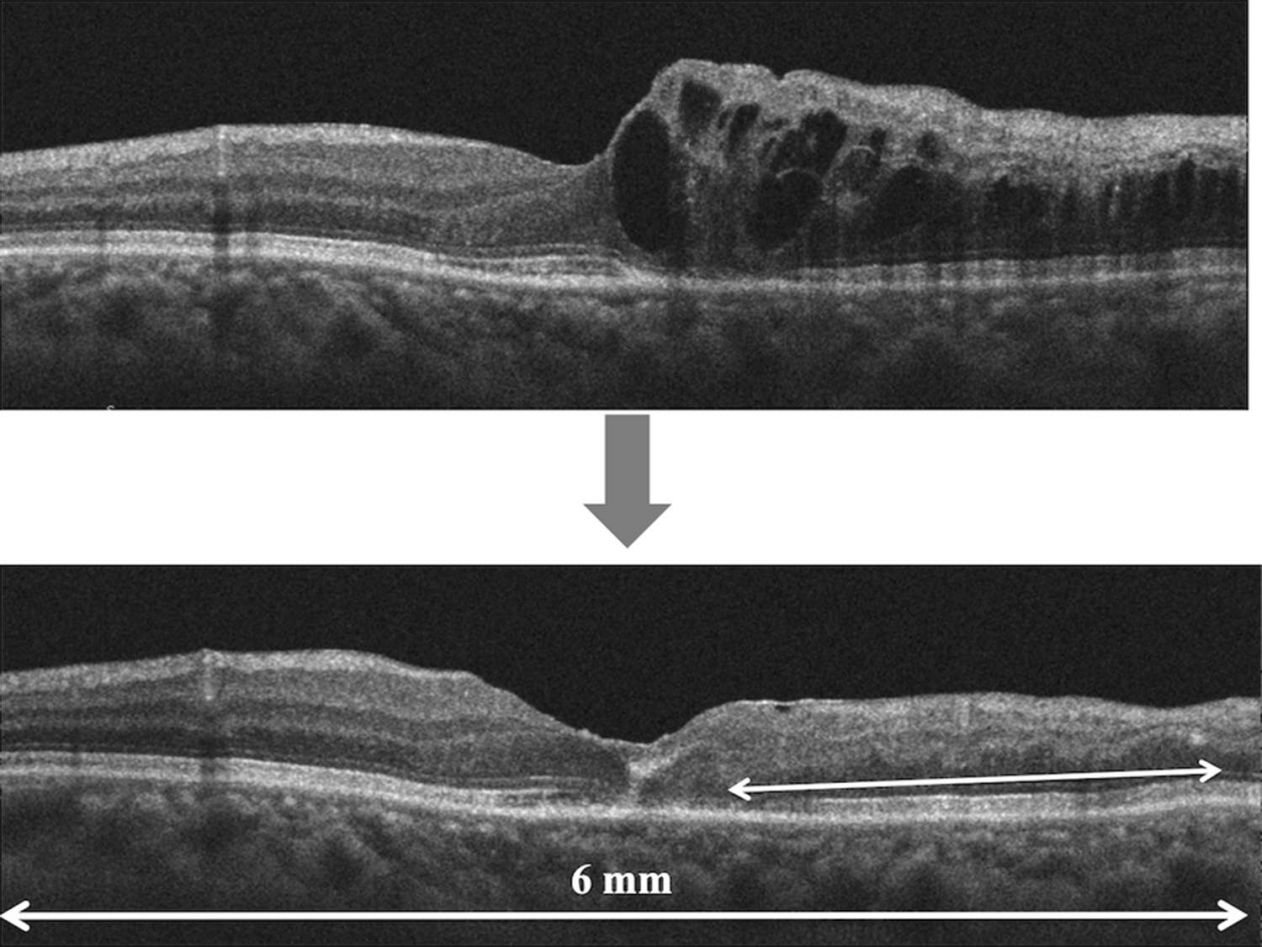
We assessed disorganization of the retinal inner layers (DRIL) at the first month after resolution of the macular edema (early DRIL) within the region of 6 mm in diameter centered on the fovea. The length of DRIL after treatment was also measured on all patients with a resolved macular edema at 12 months or around 12 months.

Retinal structure was assessed using SD-OCT. Five-line Raster Cross scans and Macular-Cube scans were performed for each eye using a commercial analytic software package (Cirrus analysis software, version 3.0; Carl Zeiss) with more than 8/10 signal strength. SD-OCT B-scans were centered across the fovea. We quantified the CRT and presence of SRD before treatment (Fig. 3 Top) on SD-OCT. At 12 months after treatment, we quantified the absence of the ELM and EZ. We evaluated the disruption of the ELM and EZ at a region of 3 mm in diameter on the vertical B-scan at the foveal center. (Fig. 3 bottom) We also assessed DRIL at the first month after resolution of the macular edema. DRIL was defined as the inability to delineate the inner retinal layer boundaries between the ganglion cell, inner plexiform layer complex, inner nuclear layer, and outer plexiform layer (Fig. 4). When the macular edema was suppressed initially, the length of DRIL was measured as "early DRIL". The length of DRIL after treatment was measured on all patients with resolved macular edema at or around 12 months. On the vertical section, we measured the length of DRIL within the region of 6 mm in diameter centered on the fovea. Two retinal specialists (YS and TM) quantified the retinal factors (including CRT, the presence of SRD, and the absence of the ELM and EZ) and measured the length of DRIL independently and the average of their measurements was used for analysis.

After diagnosis of BRVO, we treated all patients with IVR (0.5 mg Lucentis; Novartis Pharma, Tokyo, Japan) injections. All patients were evaluated monthly and treated with IVR on a PRN basis according to the following retreatment criteria: CRT of over 300 µm measured by OCT, new or persistent cystoid retinal changes, and SRD like the criteria of our previous study^10^. Clinical data were collected including age, gender, total number of injections during the 12 months, and disease duration.

The mean scores were compared and SD values were calculated for each parameter of visual function (logMAR BCVA and metamorphopsia score) and OCT measurements. One-way analysis of variance and Bonferroni tests were used to compare visual function and OCT measurements before and after treatment. Paired *t* test was run to assess the change of the length of DRIL between early phase and after treatment. The associations between visual function and OCT parameters were analyzed with the Spearman's rank correlation coefficient test. The Mann–Whitney *U* test was run to compare the visual functions between the two groups based on the presence of SRD, and the status of the EZ and ELM. All statistical analyses were performed using a commercial software package (StatView software, version 5.0; SAS, Inc, Cary, NC). All tests of associations were considered statistically significant if *P* < 0.05.
